# Resolving Anomalies in Predicting Electrokinetic Energy Conversion Efficiencies of Nanofluidic Devices

**DOI:** 10.1038/srep14725

**Published:** 2015-10-06

**Authors:** Sagardip Majumder, Jayabrata Dhar, Suman Chakraborty

**Affiliations:** 1 Department of Mechanical Engineering, Indian Institute of Technology Kharagpur Kharagpur 721302, INDIA

## Abstract

We devise a new approach for capturing complex interfacial interactions over reduced length scales, towards predicting electrokinetic energy conversion efficiencies of nanofluidic devices. By embedding several aspects of intermolecular interactions in continuum based formalism, we show that our simple theory becomes capable of representing complex interconnections between electro-mechanics and hydrodynamics over reduced length scales. The predictions from our model are supported by reported experimental data, and are in excellent quantitative agreement with molecular dynamics simulations. The present model, thus, may be employed to rationalize the discrepancies between low energy conversion efficiencies of nanofluidic channels that have been realized from experiments, and the impractically high energy conversion efficiencies that have been routinely predicted by the existing theories.

The physics of electrically modulated flows in nanofluidic channels has given rise to many apparent anomalies, primarily attributable to complex dependence of the underlying interfacial interactions on molecular scale transport mechanisms, and a complex coupling of electro-mechanics and hydrodynamics over multiple physical scales^1^,^2^,^3^. On a system level, the pertinent physico-chemical processes have often been abstracted through the introduction of adjustable tuning parameters in the concerned mathematical description, in an effort to capture the consequences of molecular level interactions from continuum considerations in an implicit fashion^1^,^4^,^5^,^6^,^7^,^8^,^9^. As a result, various aspects of hydraulic to electrical energy conversion in nanochannels remain far from being well understood, especially within the resolution of experimentally tractable scales. This deficit stems from the difficulties in capturing inter-molecular interactions through continuum based considerations, which are perceived to be appropriate only over physical scales that are substantially elevated as compared to those routinely addressed by molecular dynamics (MD) simulations.

Electrokinetics in nanofluidic channels is considered to be an emerging mechanism for converting hydraulic form of energy to electrical form^10^,^11^,^12^,^13^,^14^,^15^,^16^,^17^. An electrically neutral fluid in contact with solid substrates often develops surface charges that are counterbalanced by excess charges of opposite sign distributed in an interfacial charged layer (also known as electrical double layer; EDL)^11^,^18^,^19^,^20^,^21^. Excess counterions within the mobile part of the EDL may be forced by an externally applied pressure gradient to preferentially migrate downstream towards the end of the nanochannel, thereby developing an electrical potential difference across the channel in a dynamic environment (known as streaming potential)^22^,^23^,^24^. The resultant current, if diverted through an external resistor, may convert the supplied hydraulic form of energy to electrical form (please see the schematic in Fig. 1 for a pictorial representation of the physical scenario)^10^,^11^,^25^,^26^. Theoretically, researchers have postulated that dramatic augmentations in the concerning energy conversion efficiency may be plausible by exploiting special features of nanofluidic devices, such as interfacial slip^27^,^28^,^29^,^30^,^31^. However, in reality, experimentally obtained energy conversion efficiencies in nanofluidic channels have been observed to be significantly lower, as compared to those predicted by these theoretical simulations^7^,^32^.

Here, we attempt to resolve the above anomalies in predicting electrokinetic energy conversion efficiencies of nanofluidic devices in tune with reported experimental observations. Towards this, we devise a theoretical foundation, considering recent advancements in describing interfacial electrostatics and hydrodynamics over nanometer scales^33^,^34^, for predicting energy transfer capabilities of nanofluidic devices, which attempts to realize an effective compromise between the necessities of embedding inter-molecular interactions in standard continuum based paradigm that is unable to capture discreteness of the molecular entities explicitly, and the needs of accessing experimentally tractable scales that are often beyond the reaches of computationally involved MD simulations. We show that despite an elusively simple one-dimensional character of our model, the special features of our consideration are expected to carry no less physical content than computationally expensive molecular modeling considerations. In this manner, we report a molecularly sensitive continuum modeling paradigm that successfully predicts electrokinetic energy conversion efficiencies in nanochannels, consistent with reported experimental and MD simulation data.

Four distinctive features are embedded in our model for mimicking MD considerations in continuum-based formalism, in the context of estimating electrokinetic energy conversion efficiencies of nanofluidic devices. First, we incorporate the variations of interfacial viscosity in the streaming current estimation by invoking a suitable step function, in tune with the existence of an interfacial shear layer (~0.1 nm)^35^,^36^. Second, we capture the variations in the interfacial permittivity for deriving the charge distribution and the consequent advective and electromigration currents of the ionic species, based on the paradigm of existence of a dielectric dividing surface (DDS), having thickness of the same order as that of the shear layer^37^,^38^. A low permittivity region near the wall induces higher accumulation of counterions near the interface. Third, we consider the steric interactions due to the finite sizes of the ions in the free energy analysis resulting in the modified Poisson-Boltzmann equation. Finally, we also take into account non-electrostatic interactions^39^,^40^, within the scope of mean field approximation through the modified Poisson Boltzmann formalism. It must further be noted that the extra-thin reduced permittivity sublayer, in conjunction with a Stern layer, gives the real picture of the double layer within a very narrow confinement. This reduced permittivity sublayer is, in fact, conceptually different from the Stern layer. An explication of this fundamental consideration may be accomplished in association with the different conductivities we take into account while estimating the electromigration current through a narrow conduit^10^,^11^. At high electrolyte concentration (signifying a thin EDL), an additional surface conductance, besides the bulk conductivity, is introduced to address the conductivity of the Stern layer^10^,^41^. In order to bridge the discrepancy observed between zeta potential obtained from mobility and conductivity measurements at low concentration^42^ further, an additional *anomalous* surface conductance has also been introduced^10^,^43^. Later on, the concept of dynamic Stern Layer was introduced to address the additional anomalous conductance which characterizes the tangential ion transport very close to the substrate^44^,^45^. However, recently, MD simulation analysis have revealed the underlying mechanism of this ion transport which is attributed to the permittivity profile jump at the electrolyte-substrate interface^37^,^38^. Therefore, by virtue of the presence of excess ionic charges, the ionic conductivity close to the substrate gets elevated as compared to the bulk, obviating the unphysical need of prescribing an arbitrary conductance (anomalous surface conductance) of the immobile interfacial layer (Stern layer) of charge^44^,^45^,^46^,^47^. An effort to include such interpretations of the interfacial structural re-orientation in association with excluded size effects and non-electrostatic interaction has never been accounted in the investigation of streaming potential and energy conversion characteristics of nanofluidic channels. With the above-mentioned considerations, we establish that the physics of electrokinetic energy conversion in nanofluidic channels may be quantitatively reproduced by our simple one dimensional model, by comparing with benchmark MD simulations and reported experimental data.

## Model Description

**Figure 1:**
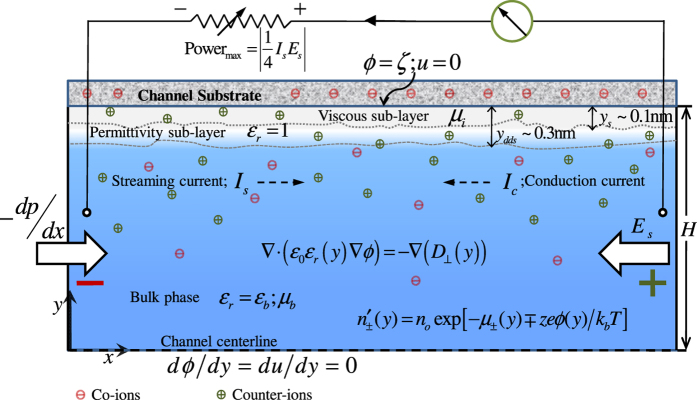
Schematic representation of the pressure driven flow of an electrolytic medium through a nanofluidic substrate. Near-wall regimes of altered viscosity and permittivity, as compared to the bulk, are schematically depicted. For illustration, the substrate is assumed to be negatively charged, so that there are abundant positive ions (counterions; shown by green colour) as compared to negative ions (coions; shown by red colour). Balance of streaming current and conduction current, in presence of the preferential distribution of counterions in the EDL as compared to coions, establishes a streaming potential. This enables the conversion of hydraulic energy to electrical energy, when the setup is connected to an external resistor. The maximum possible electrical power output through the external resistor (denoted by Power_*max*_) is proportional to the product of the streaming field potential *E*_*s*_ and streaming current *I*_*s*_ (detailed in the following sections). Credited authors: S. Majumder, J. Dhar and S. Chakraborty.

We consider a 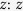 symmetric electrolyte being pumped by a driving pressure gradient 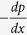 through a slit-type nanochannel of height 2*H* (see Fig. 1). The *x* axis runs parallel to the channel axis, whereas the *y* axis runs transverse to the same, with the origin being located at the channel centerline. Positive and negative ions (present with different number density distributions) in the fluid, under the action of the driving pressure gradient, get preferentially transported in the nanochannel, with a net velocity that is a cumulative effect of the fluid flow velocity and the electromigration velocity (due to forces on the ions on account of the induced axial streaming electric field *E*_*s*_). Towards this, we proceed to estimate the flow velocity field due to the imposed pressure gradient, which may be obtained by solving the momentum equation in low Reynolds number regime:


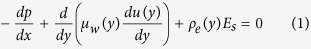


where 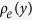 is the local volumetric charge density and *E*_*s*_ is the unknown streaming field that is induced due to the imposed flow. It must be noted that despite no application of an external electric field, an electrical body force term appears in the momentum equation due to the presence of a net charge distribution^22^,^26^, which is attributable to the induction of a streaming field. It is interesting to note here that unlike the classical formulation, we do not presume the viscosity of water (*μ*_w_) to be spatially homogeneous. Rather, we adopt a two-layer model, so that:


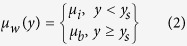


where *μ*_*i*_ is the viscosity in the interfacial sublayer region of thickness *y*_*s*_, while *μ*_*b*_ is the bulk viscosity. It must be noted that the profile in Eq. (2) is designed to reproduce the flow features as found from MD simulations for distances *y* > *y*_*s*_, since the viscosity at the adjacent interfacial subatomic layer cannot be truly described in the scope of mean-field approximation. Simulations using MD indeed reveal^34^,^35^, that there exists an interfacial sublayer of viscosity different from that of the bulk, near the wall substrate. This sublayer is not perfectly stagnant, even in the case of a hydrophilic surface, but experiences an enhanced viscosity^34^. In case of the water-hydrophilic surface pair, the width of this viscous interfacial sublayer is typically: *y*_*s*_ = 0.3 *nm* while the enhanced viscosity in this region is *μ*_*i*_ = 3*μ*_*b*_^35^. Similar trends have also been studied in other works^33^. On the contrary, for highly hydrophobic interfaces (such as diamond-water interface), the interfacial sublayer width is typically found to be *y*_*s*_ = 0.15 *nm*^2^,^35^. The corresponding decrease in apparent viscosity in the interfacial sublayer is estimated at *μ*_*i*_ = *μ*_*b*_/15^35^.

In order to obtain the velocity field and the unknown streaming potential, we need to accurately estimate the physical distribution of ions across the channel width and calculate the volumetric charge density. Towards this, we proceed by stating the free energy functional of the system and then taking its variation to derive the governing equations for this model which minimizes the free energy functional. The free energy functional must account for the finite ionic size and non-electrostatic interactions, besides the consideration of the electrostatic interaction among the ions. The basic functional form for the free energy, given by *F* = *U* − *TS* constitutes the appropriate internal energy and the entropic contribution. The internal energy *U* consists of the self-energy, electrostatic and other non-electrostatic contributions:


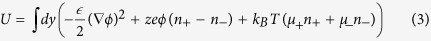


while the entropic contribution from the excluded volume effects, going beyond the ideal gas considerations^48^,^49^, reads:


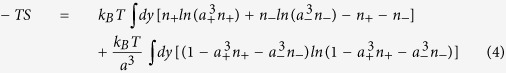


where *a*_+_(*a*_−_) is a characteristic length scale separating ionic species, which for our purpose has been assumed to represent the effective diameter of positive (negative) ions, *n*_+_(*n*_−_) represents the local ionic number density for positive (negative) ions, *ϕ* represents the electrostatic potential and *μ*_±_ is considered to be the collective non-electrostatic potential per unit thermal energy. In the above form Eq. (3), *ε* = *ε*_0_*ε*_*r*_ denotes the absolute permittivity of the medium, with *ε*_*r*_ and *ε*_0_ denoting its relative permittivity and the permittivity of free space, respectively. With the present form of the energy functional, we go beyond the traditional Poisson-Boltzmann paradigm wherein we not only relax the assumption of point charge approximation by introducing the non-ideal entropic contribution, thereby restricting the maximum ionic densities estimated near the charged interface, but also take into account the non-electrostatic interactions among the ions. The steric interactions, due to the excluded volume effects among the ions, become significant in cases of high surface charge density, large ionic radii or high electrolyte concentration^1^. The electrochemical potential 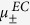 of the ions may then be obtained by the variation of the free energy functional 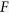 as^48^:


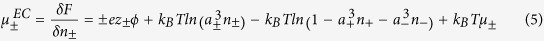


In the state of thermodynamic equilibrium, the electrochemical potential is constant and associates itself with a reference condition at the bulk with potential *ϕ* = 0 and *μ*_±_ being negligible. This results in the following expression for the electrochemical potential:


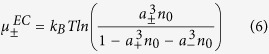


with *n*_0_ describing the number density of ions in the bulk which has been assumed to be the same for either ionic species. For the sake of simplicity without sacrificing the essential physics, we henceforth consider the same ionic size for the two ionic species in the current model *a* = *a*_+_ = *a*_−_. At steady state, the modified form of the Boltzmann distribution therefore reads:


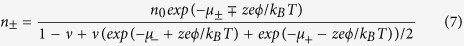


where 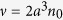 is known as the steric factor. Here, *k*_*B*_ is the Boltzmann constant and *T* is the absolute temperature. Forms resembling the ionic distribution in equation 7, accounting for the non-electrostatic effects, have been reported in numerous other theories which are derived from the grand canonical energy form and the extended DLVO theory^50^,^51^. The intricate complexities of non-electrostatic interactions between similar and dissimilar ionic species is a matter of current research. It has been proposed that a phenomenological function be considered for the non-electrostatic potential among ionic species such that the nature of non-electrostatic interactions is mimicked approximately without losing the essential physics^40^. In the present model, the form for the non-electrostatic potential function is chosen as^40^:


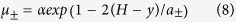


with *α* representing the strength of the non-electrostatic potential while the ion-specificity is introduced through *a*_±_. Eq. (8) ensures a higher value of *μ* near the wall where the counterion density is high, while it decays rapidly as the distance *y* is decreased towards the channel centerline. The magnitude of *α* may be interpreted as the strength of the dimensionless non-electrostatic potential which, in conjunction with the EDL electrostatic potential, dictates the ionic distribution in the EDL. Furthermore, *α* could take both positive and negative values indicating the nature of such interactions. A positive value of *α* signifies the repulsive nature of non-electrostatic interactions between ionic species while attractive interaction is brought about by a negative value of *α*. The type of channel surface also influences the choice of *α*^2^.

We now proceed to derive the governing equation by minimizing the free energy functional with respect to the electrostatic potential as 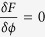, leading to the Poisson form 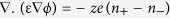, where the ionic distribution is given by the modified Boltzmann relation Eq. (7). On substituting the expressions for the ionic concentrations, we have the final form of the modified PB equation:


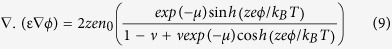


In Eq. (9), the permittivity ε does not remain constant near the fluid-substrate interface. Instead, analogous to our considerations on the existence of a jump in the viscosity profile, we again consider the existence of a jump in the perpendicular dielectric profile (ε_*r*_) very close to the wall substrate^37^,


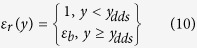


where the location of the dielectric dividing surface (*y*_*dds*_) is determined as^37^: 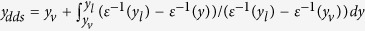, with *y*_*v*_ and *y*_*l*_ being the positions in the vapor and liquid phases respectively, while *ε*^−1^ denotes the inverse of the dielectric constant in these respective regions. The notion of the existence of a dielectric depletion layer is a direct consequence of MD simulation findings^37^,^38^, which corroborate the charge density profile variations near the charged interface. Such a jump profile can be theoretically explained by considering two interfacial effects near the channel wall^52^. Firstly, due to the accumulation of counterions near the charged electrolyte-substrate interface, solvent molecules are displaced from the interfacial region due to the finite sizes of ions. In addition to this, the reasonably high electric field in the proximity of the interface results in a preferential orientation of the solvent molecules in the case of a polar solvent. The net outcome is a reduced relative permittivity in a region close to the wall. At higher distances from the wall, the electric field is less significant due to the screening by the counterions at the wall vicinity, allowing for all possible orientations of the solvent molecules, and thus, an increased relative permittivity. Such a layer of low permittivity extends beyond the classical Stern layer and therefore needs to be accounted for in the calculation of the EDL potential distribution. Also, possible thicknesses of such permittivity depletion layers have been reported for hydrophilic and hydrophobic surfaces and the existence of a step change in permittivity has been shown^53^. The profile in Eq. (10) reproduces the potential distribution, as obtained from molecular simulations, for distances 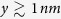 from the interface. In case of a polar solvent like water on a hydrophilic surface, the dielectric depletion layer is typically of width 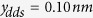, while 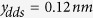 for a highly hydrophobic surface^37^.

The system of governing equations may now be brought towards a closure, only with the due consideration of an appropriate electro-neutrality condition prevailing across the channel cross-section (i.e. the net ionic current vanishes since there is no externally applied electric field). One must accordingly have: 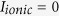. The ionic current in an electrolyte may be simply formulated as 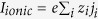 where *j*_*i*_ denotes the flux of the *i*^*th*^ ionic species. The ionic flux may be modelled in terms of the number density *n*_*i*_ as 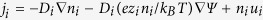
10,22, where *D*_*i*_ is the diffusivity and 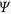 is the net potential, which in our case is given by 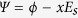. Besides the influence of solvent viscosity, finite ion size also extends its influence on the ionic diffusivity which is related to the mobility *m* per unit ionic charge through the Nernst-Einstein relation 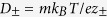 that results in the Stokes-Einstein form 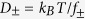
54. For the sake of simplicity, we assume 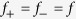 with the friction coefficient *f* being estimated from the Stokes drag force on an ion with hydration shell diameter of *a*, so that 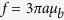
14,55. Such an assumption, where we consider the Stokes drag to hold true, may be relaxed^56^,^57^. However, again for simplicity, we do not invoke the associated complexities in this work. The net ionic current may be described as^2^: 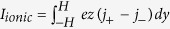 or 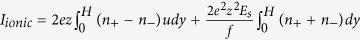. Here the fluid velocity is given by:


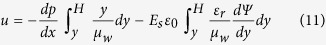


By setting 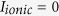, one may estimate the streaming electric field *E*_*s*_ established across the conduit.

We next non-dimensionalize our system with the aid of the following normalization parameters: 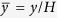, 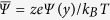, 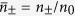, 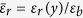, and the steric factor ν. Accordingly, the Poisson equation reads:


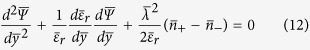


subject to the boundary conditions of symmetry at the channel centerline and a specified surface charge density (or alternatively, a specified surface potential) at the fluid-solid interface. Here 
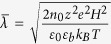
 denotes the non-dimensional Debye penetration length of the EDL. It may further be noted that the incorporation of the permittivity profile jump in the Poisson equation reflects in terms of an increased accumulation of ions near the boundary walls due to a lower dielectric constant. On the contrary, a balancing effect is given by the steric hindrance factor ν, which prevents an over saturation of the depletion layer with high ionic concentrations. The resulting induced potential distribution across the channel cross-section due to the EDL is a direct consequence of the competition between these two effects.

Utilizing the EDL potential profile obtained from the dimensionless formulation of Eq. (12), we evaluate the volumetric charge density distribution, and substitute the same in the momentum equation to get the velocity profile. The resulting solution for the velocity profile in its dimensionless form yields:


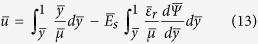


where 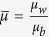, 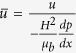 and 
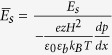
 . Recasting the dimensionless velocity as: 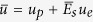, with 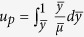, 
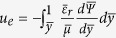
 and 
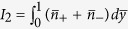
, the resulting dimensionless streaming field in a closed form expression is given by:


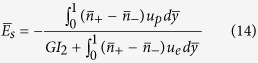


where 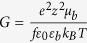.

The efficiency with which hydraulic energy is converted to electrical energy, known as electrokinetic conversion efficiency^4^,^32^,^58^,^59^,^60^, is a measure of the success of the energy transfer process in a nanofluidic channel, mediated through the establishment of the streaming potential detailed as above. The corresponding expression for the conversion efficiency is given by: 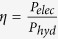, where *P*_*hyd*_ is the power supplied per unit channel length through the imposed pressure driven transport, given as 
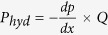
 with *Q* being the net volumetric flow rate. The maximum electrical power per unit channel length that is possible to be harvested from the induced streaming potential and the resulting conduction current equals 
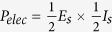
 where each quantity, namely the streaming field (*E*_*s*_) and the streaming current (*I*_*s*_), is taken at its half strength^61^,^62^. Expressed in terms of the various dimensionless parameters described as above, one finally gets:


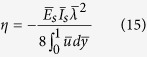


with 
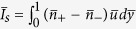
.

## Results and Discussions

For our sample model calculations reported in this work, we consider a 1:1 aqueous electrolyte with the following bulk physical properties: 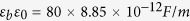, 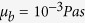, *T* = 300 *K* and the channel height 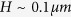 . The permittivity and viscous interfacial sublayers are considered according to the discussion in the preceding section. For a representative estimation, the steric factor is varied between 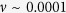 and 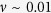 and the bulk concentration ranging from a low concentrated solution with 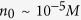 to a significantly higher concentrated electrolyte of 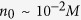 is chosen; while the value of *α* characterizing the strength of the non-electrostatic interactions is varied from −2 to 2, to illustrate the effect of a larger range of non-electrostatically interacting effects^39^,^40^. In the following figures, we have used default values of the parameters corresponding to different types of surfaces (hydrophilic or hydrophobic), as obtained from MD simulation studies of interfacial viscosity^35^ and permittivity^37^, unless stated otherwise.

### High concentration regimes

**Figure 2:**
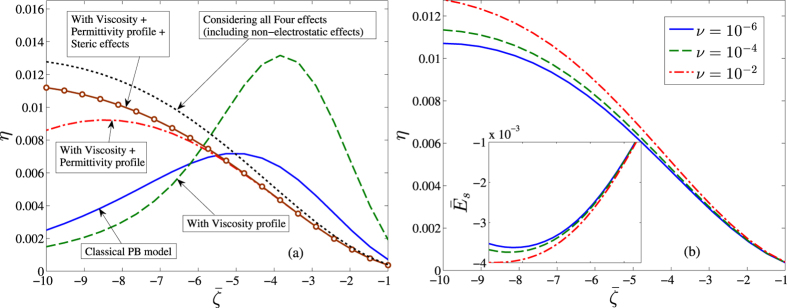
(**a**) Influence of the inclusion of various effects on the conversion efficiency *η*<inset> dimensionless streaming potential ratio 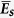 for a representative case of a hydrophobic surface, as a function of the dimensionless wall potential, 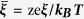  in a high concentration electrolyte (~10 *mM*) with a corresponding dimensionless Debye length scale 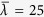 and 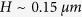. The solid-line represents the streaming potential estimation based on the classical PB model with no further effects being considered^26^, Other parameters used are: viscosity sublayer thickness 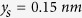, 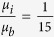, permittivity sublayer thickness 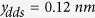, steric factor *v* = 10^−2^ and *α* = 1. (**b**) Depicts the extent of influence of varying steric factor for the similar situation as in 2a. Credited authors: S. Majumder, J. Dhar and S. Chakraborty.

**Figure 3:**
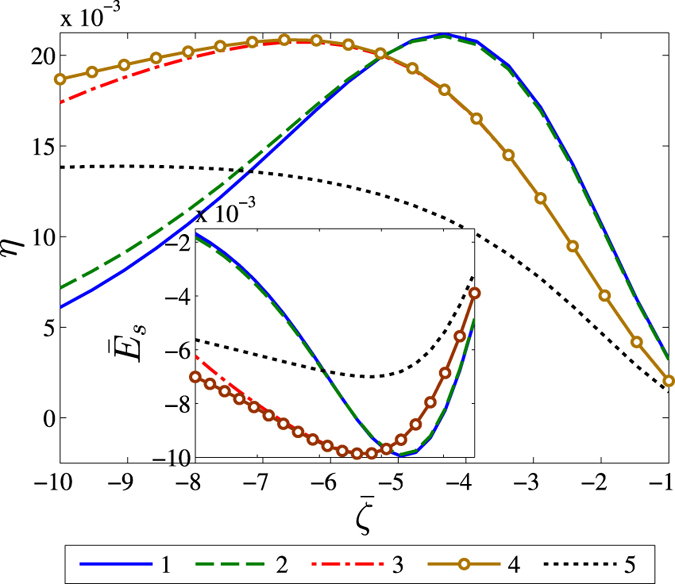
Influence of the inclusion of various effects on the conversion efficiency *η*<inset> dimensionless streaming potential ratio 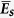 for a representative case of a hydrophilic surface, as a function of the dimensionless wall potential,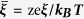. Various line numbers represent: 1) streaming potential estimation based on the classical PB model with no further effects being considered^26^, with 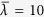 and 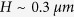; 2) inclusion of the effects of the viscous sublayer with *y*_*s*_ = 0.3 *nm* and 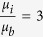; 3) considering the effect of the permittivity sublayer with *y*_*dds*_ = 0.10 *nm*; 4) inclusion of the effect of steric interactions *v* = 10^−2^; and 5) finally depicting the scenario with all four effects by adding the non-electrostatic interactions with *α* = −1.5. Credited authors: S. Majumder, J. Dhar and S. Chakraborty.

Fig. 2a depicts the effects of addition of the four factors as mentioned earlier onto the classical Poisson-Boltzmann equation at moderately high electrolyte concentrations 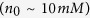. To depict the case of a higher concentration regime, the dimensionless Debye length is considered as 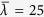, with the corresponding steric factor *v* = 0.01 signifying a strong steric interaction among the ions, especially at high wall potential. A hydrophobic surface is considered for this representative scenario. Here it can be clearly seen that the addition of each effect shows a distinctive change in the prediction of the streaming potential behavior with increasing wall 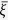 potential. The effect of steric interactions becomes prominent for high values of wall 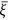 potential, where the crowding of counterions tends to be large and the concept of ionic concentration saturation becomes significant. Comparing with Fig. 3, it is clear that in different concentration regimes, the four effects produce distinctive implications and neither of these effects can be neglected in order to make a prediction across varying system conditions. Fig. 2b further shows the enhanced effect of the steric factor, at higher steric interaction zones, for a particular value of 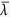. Increase in steric factor increases the streaming potential due to repulsion of the ionic charge distribution towards the bulk, thereby increasing the streaming current. As discussed above, the increase is more prominent for high wall 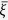 potential as well. We now proceed to discuss how these effects comparatively affect the resultant streaming field in case of a low concentrated electrolyte.

### Low concentration regimes

Fig. 3 depicts the effects of addition of the four effects mentioned earlier onto the classical Poisson-Boltzmann equation at low electrolyte concentrations 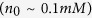. For depicting a representative scenario, we have considered a hydrophilic surface. Plot 1 in the figure depicts the results considering the classical PB based paradigm^26^, with no further effects being considered. For this case, we notice that the maximum streaming potential occurs at around 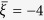. In plot 2, we add the effect of the viscous sublayer, varying the fluid rheology near the wall. This affects the streaming and conduction current in such a way that the final result is an overall increase in the induced streaming potential at high 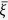. In plot 3, we add the effect of the permittivity sublayer which increases the counterion concentration near the substrate interface, due to a region of lower permittivity. Plot 4 further adds the influence of the steric effect. This results in no perceptible alterations in the solution at low surface charges, as attributable to negligible steric effects at the prevailing low counterion concentrations. However, at higher values of 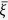, the counterion concentration tends to rise, which brings the associating steric interaction within observable limits. The result is an increase in the streaming potential and the conversion efficiency. However, this increase, which is attributed to the repelling of ionic charges more towards the centerline, is very less even for high wall potentials, due to the low concentration of the electrolyte solution and the corresponding low steric factor. Plot 5 finally includes the non-electrostatic interaction, which in this case reduces the induced streaming field and conversion efficiency. With *α* < 0, more counterions get concentrated near the substrate, eventually decreasing the streaming potential and the resulting conversion efficiency. For low wall charge, this effect reduces the streaming potential drastically; however, at higher wall charge, the effect of steric interaction becomes stronger due to higher counterion concentration, thereby hindering a drastic decrease. The influence of the non-electrostatic interaction decays with increasing wall charge, and the net result is due to the compromise between steric interactions and non-electrostatic effects at high 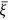 values. Summarily, the net effect is that the peaks in the conversion efficiency (and corresponding streaming potential) decrease in magnitude, while these peaks are experienced for a higher wall potential. Thus, we see that these modifications have a significant effect on the final value of the streaming potential and conversion efficiency predictions. On a broader note, a comparison with Fig. 2 clearly shows that with increase in concentration, the prominence of individual effects rise and this remains consistent with both kinds of surfaces.

### Effect of non-electrostatic potential parameter (*α*)

**Figure 4:**
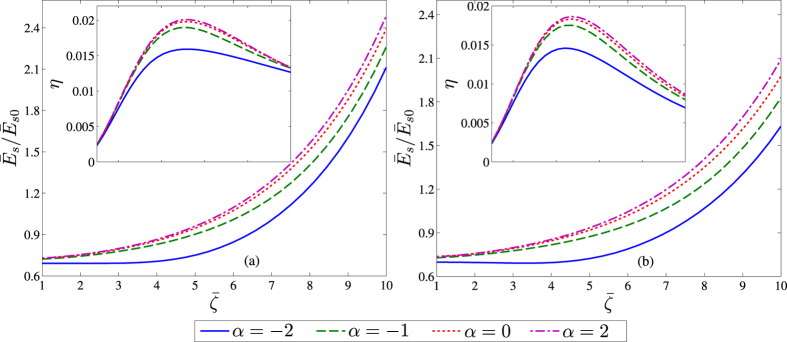
Variation of the ratio 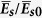 (inset: conversion efficiency *η*) as a function of dimensionless wall potential 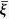, for different values of the non-electrostatic strength *α* over (a) hydrophilic and (b) hydrophobic surfaces. Other values considered are: the dimensionless Debye length 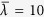 and steric factor *v* = 10^−4^. 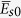 is the dimensionless streaming potential field obtained from the classical PB description. Credited authors: S. Majumder, J. Dhar and S. Chakraborty.

We next attempt to assess the implication of the parameter *α*. Fig. 4 describes the ratio 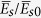 as a function of 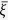 for different values of *α*, corresponding to: a) a hydrophilic surface (with typical contact angle of 80°), and b) a hydrophobic surface (with typical contact angle of 140°)^36^, where 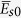 is the streaming potential field from the solution of the classical PB equation with no existence of sublayers assumed. The inset of Fig. 4 shows the corresponding variations in the conversion efficiency. We observe that the induced streaming potential and the resulting conversion efficiency are slightly less for hydrophobic surfaces in comparison to that corresponding to hydrophilic surfaces for similar values of *α*. This observation has also been shown in other studies depicting two types of surfaces^63^. Although, in reality, the presence of hydrophobic surface does enhance the efficiency to some extent as it corresponds to the case of *α* > 0 (which points to a higher efficiency compared to the hydrophilic cases characterized by *α* < 0), our model does not predict giant augmentations in efficiency in such scenarios as previously predicted in some theoretical studies^27^,^31^. For *α* < 0, we see that the induced streaming potential is less. This may be attributed to the fact that a negative non-electrostatic potential attracts the ionic species (here the counterions) towards the wall, leading to a lower streaming field. On the contrary, when *α* > 0, the induced streaming field is higher since the counterions are generally repelled away from the wall more effectively and get distributed towards the bulk region. Consequently, a higher conversion efficiency is achieved when compared to the previously calculated reference of 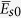. One more observation pertaining to the current figure corresponds to the case when 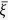 is less. In this situation, the effect of the non-electrostatic interaction among the ions is not so pronounced, as compared to the effects due to the presence of the wall-adjacent sublayers. Thus, a ratio lower than unity is obtained.

### Comparison with other models

**Figure 5:**
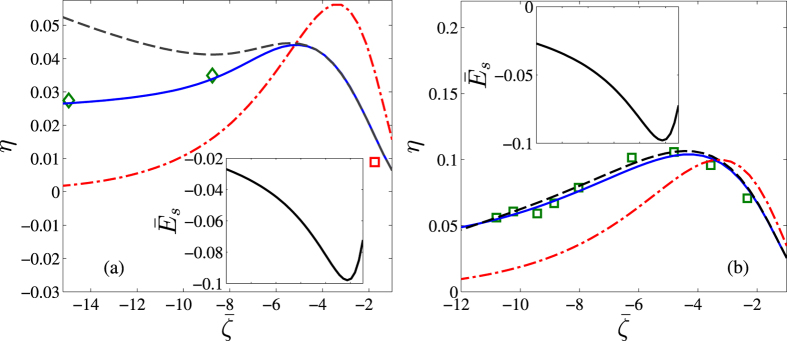
Electrokinetic Conversion efficiency against 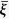, estimated from different modeling considerations, for (a) hydrophilic surface and (b) hydrophobic surface. <Inset> Corresponding dimensionless streaming potentials predicted from the present model. The solid lines represent the predictions from the present model, with all the addressed effects taken into consideration. The dashed lines represent model predictions where only the viscosity jump is replaced by Navier slip based hydrodynamic boundary condition and keeping rest of the conditions same (see main text below for details). The dash-dot lines represent the predictions from the classical PB formalism. The markers represent MD simulation data. (**a**) The diamond markers corresponds to data reported in ref. 3, whereas the square marker corresponds to data reported in ref. 64 Other values chosen are *α* = −1.5, *v* = 10^−4^, *y*_*dds*_ = 0.10 *nm, y*_*s*_ = 0.30 *nm*, 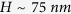 and 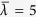. (**b**) The MD results (square markers) were obtained from a single simulation (ref. 7). Other values chosen are *α* = 1, *v* = 10^−4^, *y*_*dds*_ = 0.12 *nm, y*_*s*_ = 0.15 *nm*, 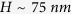 and 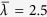. Credited authors: S. Majumder, J. Dhar and S. Chakraborty.

Fig. 5 describes the values of the energy conversion efficiencies considering different modeling approaches, as a function of the variation of the surface charge. It gives an overall comparison of various methods of estimation of the electrokinetic conversion efficiency, in perspective of reported MD simulation data. For the dashed line, plots of the electrokinetic conversion efficiencies are obtained by the usual combination of all the above effects except employing the near wall viscosity jump where instead a slip boundary condition based approach is used. We have derived the streaming field by incorporating a slip length (*b*) (typically obtainable from MD data) at the wall through the velocity profile. For the specific results reported here, we have taken 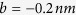 for the hydrophilic surface, while 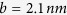
35 for the hydrophobic surface. The corresponding non-dimensional streaming potential field then reads 
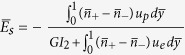
, where the non-dimensional pressure driven and electrical components of the velocity field 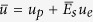 are given by 
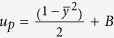
 and 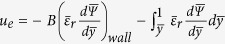 respectively, *B* = *b*/*H* being the dimensionless slip length. For the MD simulation results, we have used the Grahame equation 
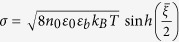
, to obtain the equivalent dimensionless zeta potential 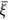 from the surface charge density, *σ*. The remaining dash-dot line represents the prediction using the classical PB based approach. From Fig. 5, it is evident that considering viscous and permittivity sublayers of the order of nanometers using the present model, a close prediction of the MD simulation results may be achieved for a wide range of surface potentials, using the present continuum based theory. In Fig. 5a, we see that results from the present model (solid line) show quite accurate agreement with the molecular simulation data (markers), besides exhibiting an efficiency peak which is commonly encountered. The slip-based model (instead of considering near-wall viscosity variations) along with the dielectric profile (dashed line), however, cannot predict the MD data especially at higher wall potentials (see Fig. 5a), corresponding to hydrophilic surfaces. If one neglects the presence of the added effects altogether, the model (dot-dash line) completely fails to reproduce the MD simulation data. In Fig. 5b, we again notice that the estimation from our present model (solid line) gives very close predictions compared to the MD simulation data (square markers) obtained from^7^, corresponding to hydrophobic substrates. The present modeling considerations, in conjunction with the slip based paradigm (instead of considering near-wall viscosity variations), also captures the MD trends quite effectively for this case, consistent with the practicalities of slipping hydrodynamics over hydrophobic surfaces. However, simulations with the classical PB formalism appear to fail severely in capturing the MD predictions for this case as well.

### Experimental Perspective

We next attempt to validate our findings with reported experimental data. The dimensional parameters remain the same, unless specifically stated based on the particular experimental considerations. For all cases, we have considered the strength of the non-electrostatic potential as *α* = −1 (which closely captures MD information over a wide range of physical data^2^). In the first experimental comparison (based on^32^), the channel height is taken as 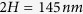, 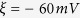, and 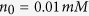. The resulting conversion efficiency is obtained as 5.4% which is close to the experimental finding which predicts the maximum efficiency cannot exceed the value ~6%. In theoretical analysis on these experiments as reported earlier, significantly elevated values of the conversion efficiency were, however, predicted^4^. In another work^59^, with 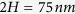, 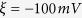 and 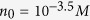, the present model predicts a maximum conversion efficiency of 3.4% which is close to the reported maximum of ~3.2%, while reported theories have predicted an efficiency of the order of 7%^58^. Validating a further experimental situation^62^, where a porous plug, with pore size in the order of 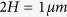, was used containing 1 *mM* concentrated solution and inducing a wall potential of 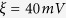, we obtain a conversion efficiency much less than 1% as has been reported in that study. Thus, in general, we note that the present formulation achieves a reasonable degree of improvement in predicting the electrokinetic conversion efficiency as compared to the predictive capabilities of the existing continuum based paradigms, and is applicable over a wide range of surface potentials. The heuristic parameter *α* as represented here, was used to elucidate the discrepancy between experimentally measured capacitance data and that obtained from Guy-Chapman model by Bonthius and Netz^2^. Here we have chosen values of *α* that are consistent with MD simulations results corresponding to a particular channel surface type for evaluating the non-electrostatic potential as employed in previous studies^2^. However, more accurate data for the ionic hydration shell size, non-electrostatic interaction strength and influence of surface properties on the viscous and permittivity regimes will eventually lead to more accurate theoretical predictions of the electrokinetic energy conversion efficiencies. Nevertheless, considerations from the present study may act as a fundamental conceptual basis towards addressing those needs.

**Figure 6:**
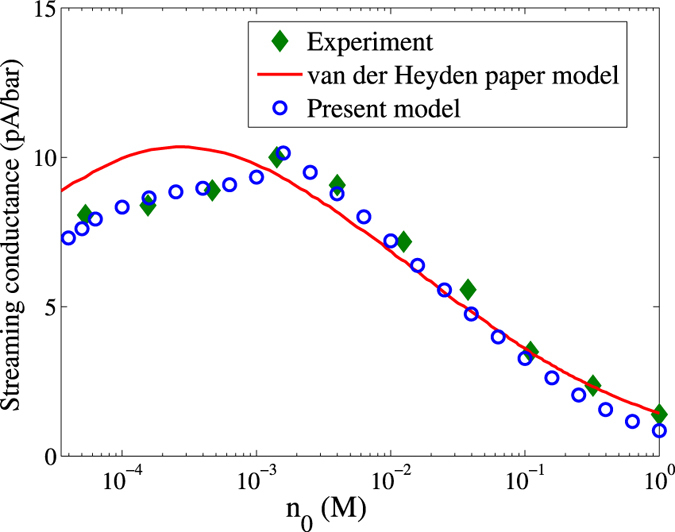
Comparison of the present model predictions (denoted by circular markers) with experimental findings 32 (denoted by diamond markers) of the streaming conductance for various electrolyte concentrations. The solid line represents the predictions of the theoretical model used in the same experimental study. Credited authors: S. Majumder, J. Dhar and S. Chakraborty.

Fig. 6 depicts the comparison of the present model predictions against reported experimental findings^32^ for the streaming conductance at various electrolyte concentrations. The circular markers denote predictions from the model developed in the present study, the diamond markers represent the experimental results while the solid line represents the predictions of the theoretical model used in the same experimental study. We have considered the channel half-height of 140 *nm* for the validation of our model. The wall 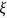 potential is estimated from the corresponding concentration values by employing the expression: 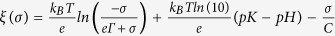, in conjunction with the Grahame equation, where *σ* is the surface charge density, *Γ* is the surface density of chargeable sites, *pK* is the dissociation equilibrium constant, and *C* denotes the Stern layer capacitance^32^. Using these considerations, it can be observed that the present model is capable of reproducing the experimental trends to a satisfactory extent over a wide range of ionic concentrations, considering *α* = −1.

## Conclusions

In this work, we have attempted to develop a simple continuum based theoretical model that is capable of rationalizing the discrepancies between abnormally large values of electrokinetic energy conversion efficiencies that have been routinely predicted by theoretical studies and significantly lower values of the same that have so far been realized from experimental practice. We have attempted to devise our model as an effective compromise between the needs of improvising the existing continuum based models with synthesized molecular-level information, and the needs of accessing experimentally tractable physical scales that are truly beyond the capabilities of computationally-intensive MD simulations. By validating with benchmark MD data as well as with reported experimental results, we have established that despite its simplicity and one-dimensional nature, our modeling consideration is consistent with the interfacial physico-chemical phenomena occurring over the disparate spatio-temporal scales. Our modeling paradigm, in effect, may act as a reliable design basis for predicting the electrokinetic energy conversion capabilities of nanofluidic devices, with a highly reasonable compromise between physical consistency and computational economy.

## Additional Information

**How to cite this article**: Majumder, S. *et al.* Resolving Anomalies in Predicting Electrokinetic Energy Conversion Efficiencies of Nanofluidic Devices. *Sci. Rep.* **5**, 14725; doi: 10.1038/srep14725 (2015).
